# Tumour growth rate of carcinoma of the colon and rectum: retrospective cohort study

**DOI:** 10.1002/bjs5.50355

**Published:** 2020-09-30

**Authors:** J. R. Burke, P. Brown, A. Quyn, H. Lambie, D. Tolan, P. Sagar

**Affiliations:** ^1^ John Golligher Colorectal Surgery Unit Leeds UK; ^2^ Department of Clinical Radiology, Gastrointestinal and Abdominal Radiology, St James's University Hospital, Leeds Teaching Hospitals NHS Trust Leeds UK; ^3^ Leeds Institute of Biomedical and Clinical Sciences St James's University Hospital Leeds UK

## Abstract

**Background:**

The growth pattern of colorectal cancer is seldom investigated. This cohort study aimed to explore tumour growth rate in colorectal cancers managed non‐surgically or deemed not resectable, and to determine its implication for prognosis.

**Methods:**

Consecutive patients with colonic or rectal adenocarcinoma were identified through the colorectal multidisciplinary team database at Leeds Teaching Hospitals NHS Trust over a 2‐year interval. Patients who received no treatment (surgery, stenting, colonic defunctioning procedures, chemotherapy, radiotherapy) and who underwent CT twice more than 5 weeks apart were included. Multidetector CT/three‐dimensional image analysis was performed independently by three experienced radiologists.

**Results:**

Of 804 patients reviewed, 43 colorectal cancers were included in the final analysis. Median age at first CT was 80 (73–85) years and the median interval between scans was 150 (i.q.r. 72–471) days. An increase in T category was demonstrated in 31 of 43 tumours, with a median doubling time of 211 (112–404) days. The median percentage increase in tumour volume was 34·1 (13·3–53·9) per cent per 62 days. The all‐cause 3‐year mortality rate was 81 per cent (35 of 43) with a median survival time of 1·1 (0·4–2·2) years after the initial diagnostic scan. In those obstructed, the relative risk of death from subsequent perforation was 1·26 (95 per cent c.i. 1·07 to 1·49; *P* = 0·005).

**Conclusion:**

This study documented a median doubling time of 211 days, with a concerning suggestion of tumour progression, which has implications for the current management standard.

## Graphical abstract

Few studies have evaluated the growth pattern of colorectal cancer. Forty‐three patients who underwent CT twice more than 5 weeks apart were included over a 2‐year interval. The median doubling time was 211 days, with a concerning suggestion of tumour progression. This has implications both under the current 62‐day UK standard and following the rapid emergence of COVID‐19 and its global impact on surgical cancer care.

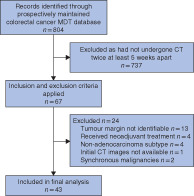

Tumor volume changes in colorectal cancer

## Abstract (es)

**Antecedentes:**

El patrón de crecimiento del CRC (*colorectal cancer*, CRC) ha sido poco investigado. El objetivo de este estudio de cohortes fue explorar la tasa de crecimiento tumoral en los pacientes con CRC no tratados quirúrgicamente o con tumores irresecables para determinar su valor pronóstico.

**Métodos:**

Los pacientes consecutivos con adenocarcinoma de colon o recto se identificaron a partir de la base de datos del equipo multidisciplinario colorrectal del “Leeds Teaching Hospitals NHS Trust” durante un período de 2 años. Se incluyeron los pacientes que no recibieron tratamiento (cirugía, colocación de endoprótesis, procedimientos de desfuncionalización del colon, quimioterapia, radioterapia), en los que se obtuvieron tomografías computarizadas con > 5 semanas de diferencia. El análisis de imágenes TC/3D multidetector fue realizado de forma independiente por tres radiólogos expertos.

**Resultados:**

De los 804 pacientes revisados, 43 CRCs se incluyeron en el análisis final con una mediana de 150 días (rango intercuartílico, *interquartile range*, IQR: 72‐471) entre los escáners. La mediana de edad en el primer escáner era de 80 años (IQR: 73‐85). En 31 (72%) casos, se demostró un aumento del estadio TNM del tumor, con un tiempo medio de duplicación del tamaño tumoral de 211 días (IQR: 112‐404). La mediana de aumento porcentual del volumen del tumor era de un 34% cada 62 días (IQR: 13,3‐53,9). La mortalidad por cualquier causa a los 3 años fue del 81% (35/43), con una mediana de supervivencia de 1,1 años (IQR: 0,4‐2,2) desde el escáner inicial diagnóstico. El riesgo relativo de mortalidad como resultado de la obstrucción intestinal y perforación subsiguiente era de 1,26 (i.c. del 95% 1,07‐1,49, *P* < 0,01).

**Conclusión:**

Este estudio documentó una mediana de tiempo de duplicación del tamaño del tumor de 211 días, así como datos preocupantes de la progresión del tumor que podrían tener repercusión en el tratamiento estándar actual.

## Introduction

Colorectal cancer is the third most common cancer diagnosed in men and second most common in women internationally, with an increasing incidence in those aged less than 50 years^1^, ^2^. Screening guidelines vary worldwide^3^; paired faecal occult blood testing and endoscopic investigation have been shown to increase the detection rate of asymptomatic cancers. As a result, mortality has decreased by 30 per cent in participants, but with an increase in cancer numbers requiring operation and patients therefore waiting longer for surgery^4^, ^5^, ^6^, ^7^, ^8^, ^9^, ^10^, ^11^. There is, however, a paucity of evidence surrounding the rate at which colorectal tumours grow once they are established.

Tumour growth and invasion is paramount to oncological outcomes as cancer advances through multiple distinct stages in its transition from indolent to invasive disease^12^, ^13^. In the UK, guidelines to reduce the time that patients wait for cancer care and specific time standards from referral to first definitive treatment were introduced in 2009, and are now enshrined in the National Health Service (NHS) constitution. Although introduced with a laudable aim, the current 62‐day standard does not have a scientific basis^14^. To date, evaluation of colorectal cancer growth patterns is limited, predominantly because of the inherent ethical issues associated with a prospective longitudinal study of leaving diagnosed colorectal cancer untreated without clinical justification.

Few studies have investigated the growth patterns of colorectal tumours endoscopically, and even fewer radiologically^15^, ^16^, ^17^, ^18^. A serial double‐contrast barium enema study^19^ conducted in 1963 demonstrated a median tumour doubling time of 620 days. Since then, only one study has assessed the growth of colorectal tumours using modern imaging techniques (*Table* *S1*, supporting information)^20^, ^21^, ^22^, ^23^, ^24^, ^25^.

Current treatment strategies are determined by the stage of disease, associated co‐morbidities, likely prognosis and patient preference. Staging for colorectal cancer is dependent on cross‐sectional imaging, which is pivotal in the assessment of recurrence and metastatic disease in patients who have received treatment^26^, ^27^, ^28^, ^29^. Use of imaging to help stratify patients is vitally important as neoadjuvant and adjuvant treatment options continue to advance. There is a growing demand for accurate quantification of tumour growth to optimize the timing of surgery or to assess the potential benefits of non‐surgical therapy^30^, ^31^, ^32^, ^33^, ^34^, ^35^. Precise quantification of tumour growth would have a significant impact for patients who choose non‐surgical therapy in terms of both prognosis and informed consent. However, this also has medicolegal implications in terms of missed cancers, and in the consequences of a delay to standard investigations and surgical care.

The aim of this study was to measure tumour growth rates in a subgroup of untreated colorectal cancers, to provide prognostic value in patients with tumours managed non‐surgically or deemed not resectable, and to determine the implications of delay to diagnosis.

## Methods

Consecutive patients with colonic or rectal adenocarcinoma treated between 1 January 2016 and 31 December 2017 were identified through the institutional colorectal multidisciplinary team (MDT) database at Leeds Teaching Hospitals NHS Trust, and through a prospectively maintained radiology discrepancy and educational database at the same institution.

Patients were included if they had undergone CT twice at least 5 weeks apart (at least 1 within the tertiary referral centre) between April 2009 and September 2018 (any indication for repeat CT was considered), and during the same interval had received no tumour treatment: surgery (including stent insertion or colonic defunctioning procedures), chemotherapy, radiotherapy or any combination. Exclusion criteria were: patients with synchronous malignancies (those with altered tumour biology such as patients receiving systemic treatments); inability to identify the tumour or tumour margins (very small tumours or where artefact rendered CT image interpretation impossible); and non‐adenocarcinoma subtype.

### Data collection

Electronic clinical and radiological databases were used to obtain patient demographic details, clinical history, treatment data, clinical outcome and follow‐up duration. Electronic records included the institutional radiology information system (Computerised Radiology Information System; Healthcare Software Systems, Mansfield, UK) and the oncology electronic patient record system (Patient Pathway Manager; EHR Development Team, Leeds Teaching Hospitals NHS Trust, Leeds, UK). Mortality was determined through hospital electronic records, which are paired with community and bereavement systems.

Prospective consent was obtained from all patients at the time of imaging for use of anonymized CT imaging data in research and service development projects. Formal ethics committee approval was waived for this study, which was considered by the institutional review board to represent evaluation of a routine clinical service.

### Imaging acquisition and reconstruction

All patient examinations were re‐examined retrospectively by three consultant radiologists. Multidetector CT (Siemens, Munich, Germany; GE Healthcare, Waukesha, Wisconsin, USA) was used with full abdominal and pelvic acquisition in a single breath hold. The scan was acquired at 120 kV, 80 mA, tube rotation time 0·5 s per rotation, and pitch 6. Images had a collimation and slice thickness of at least 5 mm (median 3 mm) but were often acquired at 1 mm slice thickness. All images were acquired using iterative reconstruction. Image analysis was performed on the thinnest slice thickness available. Where iodinated intravenous contrast material was administered, the image was acquired in the portal venous phase after administration of 100 ml contrast.

### Image segmentation

Postprocessing and image analysis included three‐dimensional lesion measurement using Advanced Workstation software (AW 3.2; GE Healthcare). The primary tumour was delineated using a semiautomated technique based on thresholding of the tumour using the lesion density (seeding around a set Hounsfield unit) within the tumour. This was then adjusted manually to outline the peripheral contours of the tumour on each image. This was performed in the axial plane with correlation on coronal and sagittal plane imaging. The tumour volume was calculated automatically by the software by multiplication of the cross‐sectional area by the slice thickness. This process was repeated independently on the baseline and follow‐up images by three experienced clinical radiologists (with 3, 10 and 15 years of experience of gastrointestinal CT), with agreement by consensus. Simultaneous tumour staging was documented using the TNM classification, eighth edition^36^.

**Fig. 1:**
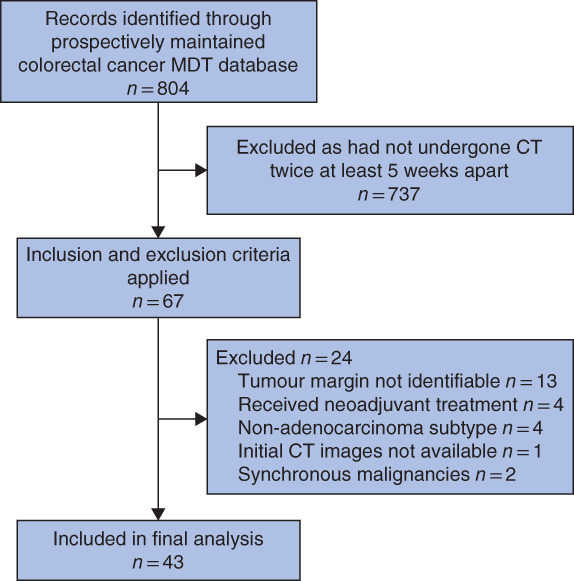
Study flow chart MDT, multidisciplinary team.

### Growth calculations

Length growth rate (mm/day) = (diameter_follow‐up_ – diameter_baseline_)/(time_follow‐up_ – time_baseline_).

Length growth rate % of baseline size = ((diameter_follow‐up_ – diameter_baseline_)/diameter_baseline_) × 100.

% increase in tumour length per 62 days = (tumour length growth rate % of baseline size/(time_follow‐up_ – time_baseline_)) × 62.

Volume growth rate (cm^3^/day) = (volume_follow‐up_ – volume_baseline_)/(time_follow‐up_ – time_baseline_).

Volume growth rate % of baseline size = ((volume_follow‐up_ – volume_baseline_)/volume_baseline_) × 100.

% increase in tumour volume per 62 days = (volume growth rate % of baseline size/(time_follow‐up_ – time_baseline_)) × 62.

Tumour doubling time = (ln2(time_follow‐up_ – time_baseline_))/(ln2(volume_follow‐up_ – volume_baseline_)).

### Statistical analysis

All data were tabulated in Microsoft Excel® (Microsoft, Redmond, Washington, USA). Continuous data are presented as median (i.q.r.). Subgroups were compared by means of Kruskal–Wallis ANOVA. All statistical analysis comparing tumour sizes, stages and growth rates was completed using SPSS® version 23 (IBM, Armonk, New York, USA).

## Results

During the study interval, 804 patients were referred to the colorectal MDT, of whom 67 met the inclusion criteria and all reviewed examinations were deemed of satisfactory quality. Twenty‐four were subsequently excluded, leaving 43 tumours for inclusion in the analysis (*Fig*. *1*). Median patient age at the time of first CT was 80 (i.q.r. 73–85) years; there were 23 women and 20 men. No patients included in the final analysis had any previous diagnosis of underlying bowel pathology.

### Tumour site and stage

Tumour site and TNM stage are summarized in *Tables* *1* and *2*. Ten tumours had radiological features suggestive of mucinous adenocarcinoma. An increase in T category was observed in 31 on the follow‐up CT, a median of 150 (72–471) days after the first scan (*Table* *3*). The N category changed in 25 patients, extramural venous invasion in 14 patients and metastatic invasion in 11. Thirty‐three of 43 tumours were non‐mucinous (15 right‐sided and 18 left‐sided). Of the ten mucinous tumours, six were right‐sided and four left‐sided.

**Table 1:** Tumour site and cohort demographics

Tumour site	No. of patients	Sex ratio (M : F)	Age (years)*
Caecum	8	3 : 5	80 (72–85)
Ascending colon	7	1 : 6	82 (77–85)
Transverse colon	6	3 : 3	80 (74–86)
Descending colon	3	2 : 1	60 (55–87)
Sigmoid colon	13	8 : 5	78 (74–87)
Rectum	6	3 : 3	76 (57–82)
Total	43	20 : 23	80 (73–85)

* Values are median (i.q.r.).

**Table 2:** TNM staging at initial and repeat CT

	Initial diagnostic CT (*n* = 43)	Repeat CT (*n* = 43)
**T category**		
T1	0	0
T2	13	1
T3a	6	4
T3b	11	8
T3c	7	7
T3d	0	3
T4a	2	13
T4b	4	7
**N category**		
N0	24	15
N1	14	16
N2	4	12
Nx	1	0
**M category**		
M0	35	24
M1	8	19
Mx	0	0
**EMVI**	18	32

EMVI, extramural vascular invasion.

**Table 3:** Growth calculation results and subgroup analysis

	Interval between 1st and 2nd CT (days)	Change in tumour length (mm)	Specific length growth rate (%)	Change in tumour volume (cm^3^)	Volume increase from baseline (%)	Tumour doubling time (days)	Absolute growth per 62 days (mm)	Volume growth per 62 days (cm^3^)
**All tumours (*n* = 43)**	150 (72–472)	9·5 (2·0–22·3)	23·5 (3·4–53·5)	18·3 (7·9–48·0)	102·2 (43·1–292·4)	211 (112–404)	2·23 (0·86–4·89)	6·85 (1·68–13·58)
**Anatomical location**								
Right colon (*n* = 21)	335 (115–650)	16·0 (0·5–31·0)	41·0 (0·4–68·2)	24·7 (7·6–72·1)	175·2 (69·0–462·8)	211 (100–598)	1·75 (0·28–4·93)	5·21 (1·53–12·85)
Left colon (*n* = 16)	142 (3–326)	7·5 (0–15·8)	15·7 (0·04–38·2)	14·1 (0·03–35·9)	55·8 (0·2–204·1)	227 (179–409)	2·87 (2·62–5·03)	7·01 (1·74–11·71)
Rectum (*n* = 6)	280 (59–983)	15·0 (1·3–26·0)	31·6 (2·5–66·2)	31·6 (2·5–66·1)	26·9 (7·7–57·9)	142 (99–343)	2·34 (0·49–5·63)	8·15 (2·24–13·63)
*P* *	0·276	0·510	0·418	0·498	0·059	0·609	0·698	0·867
**Mucinous type**								
Yes (*n* = 10)	227 (96–379)	16·5 (1·8–32·0)	36·1 (1·1–57·6)	63·8 (22·6–152·6)	214·1 (67·2–405·4)	168 (105–265)	2·91 (0·99–6·87)	13·86 (8·02–29·46)
No (*n* = 33)	167 (96–577)	9·0 (2·0–20·0)	20·0 (3·7–51·4)	15·5 (8·8–32·8)	81·6 (38·2–286·3)	235 (117–434)	2·02 (0·85–4·72)	6·20 (1·53–8·70)
*P* *	0·367	0·369	0·274	0·008	0·162	0·181	0·273	0·003
**T category change**								
Progression (*n* = 31)	182 (114–518)	10·0 (2·0–22·0)	23·3 (3·4–51·1)	19·5 (9·7–60·0)	119·0 (46·9–327·1)	204 (123–419)	1·75 (0·84–4·89)	6·85 (1·68–13·58)
Stable (*n* = 12)	121 (90–362)	11·0 (2·3–32·6)	23·2 (3·0–63·4)	21·0 (7·1–45·8)	63·4 (18·5–286·2)	237 (110–401)	2·57 (1·93–5·23)	7·13 (1·43–21·97)
*P* *	0·118	0·391	0·446	0·412	0·120	0·443	0·219	0·447
**T category progression**								
T2 → T3/T4 (*n* = 12)	485 (307–957)	15·0 (0·5–28·3)	44·1 (1·2–68·0)	30·1 (10·5–56·8)	269·1 (133·0–821·4)	203 (151–581)	0·91 (0·23–2·62)	2·92 (1·51–9·76)
T3 → T4 (*n* = 19)	136 (105–337)	9·5 (2·8–19·0)	17·2 (5·1–42·0)	46·2 (9·3–53·8)	71·5 (32·5–226·1)	199 (105–335)	2·57 (1·04–8·69)	7·78 (4·81–14·38)
*P* *	*P*= 0·002	0·251	0·143	0·208	0·002	0·201	0·035	0·059
**N category change**								
Progression (*n* = 25)	204 (100–505)	10·0 (2·5–26·5)	23·3 (3·1–54·8)	27·4 (10·3–64·5)	104·7 (36·8–487·4)	211 (117–363)	2·81 (0·90–5·17)	8·95 (2·04–14·56)
Stable (*n* = 18)	152 (96–447)	9·0 (1·3–18·0)	21·1 (2·3–50·0)	14·6 (7·9–27·4)	80·7 (44·6–255·3)	208 (112–629)	1·56 (0·55–5·13)	5·70 (1·41–8·07)
*P* *	0·387	0·222	0·354	0·068	0·111	0·332	0·288	0·274
**M category change**								
Progression (*n* = 11)	155 (66–635)	18·0 (3·0–32·0	41·9 (5·7–60·0)	18·1 (8·5–57·0)	84·5 (33·2–550·9)	172 (112–374)	3·12 (1·07–8·27)	7·98 (5·21–15·71)
Stable M0 (*n* = 25)	331 (124–512)	12 (12·5 –23·8)	30·0 (3·0–62·8)	27·4 (11·2–67·9)	202·8 (55·0–388·7)	221 (155–401)	2·23 (0·87–4·04)	6·61 (1·99–13·89)
Stable M1 (*n* = 7)	108 (91–150)	3·0 (0–8·0)	7·7 (2·6–20)	9·1 (1·0–23·7)	27·3 (3·3–93·9)	213 (60–1091)	1·24 (0·26–4·56)	6·26 (0·41–10·56)
*P* *	0·082	0·086	0·101	0·093	0·078	0·842	0·457	0·399

Values are median (i.q.r.).

* Kruskal–Wallis ANOVA.

### Tumour volume changes

Growth calculation results are shown in *Table* *3*. For all tumours, the median change in tumour length was 9·5 (2·0–22·3) mm; expressed as a percentage of the baseline tumour size, the specific growth rate (length) was 23·5 (3·4–53·5) per cent. The median change in tumour volume was 18·3 (7·9–48·0) cm^3^, which equated to a percentage increase from the baseline volume of 102·2 (43·1–292·4) per cent; thus, the tumour volume approximately doubled in a median of 150 (72–472) days. The median absolute growth per 62‐day period was 2·23 (0·86–4·89) mm and the median volume growth was 6·85 (1·68–13·58) cm^3^ over 62 days. This corresponded to a median percentage increase in tumour length of 5·5 (1·8–9·2) per cent over 62 days and a median percentage increase in tumour volume of 34·1 (13·3–53·9) per cent over 62 days. The median tumour doubling time was 211 (112–404) days.

In subgroup analysis, a greater percentage volume increase from baseline was observed for right‐sided colonic tumours *versus* left‐sided colonic and rectal tumours: median 175·2 (69·0–462·8), 55·8 (0·2–204·1) and 26·9 (7·7–57·9) per cent respectively; however, the difference was not statistically significant (*P* = 0·059) (*Table* *3*). There was a significant association between mucinous subtype and increase in median volume growth. Mucinous tumours had double the growth rate of non‐mucinous tumours per 62 days: median 13·86 (8·02 to 29·46) *versus* 6·20 (1·53–8·70) cm^3^ (*P* = 0·003). The median absolute growth per 62 days was greater for more advanced tumours that progressed from T3 to T4 than for lesions that progressed from T2 to T3/T4: 2·57 (1·04–8·69) *versus* 0·91 (0·23–2·62) mm (*P* = 0·035).

### Mortality

The all‐cause 3‐year mortality rate was 81 per cent (35 of 43), with a median life span of 1·1 (0·4–2·2) years after the initial diagnostic CT; the cause of death was a direct result of bowel obstruction and subsequent perforation in five patients. Eight patients were alive at the study conclusion, with a median follow‐up time of 3·4 (2·1–6·1) years after the initial diagnostic CT. Seven patients had the initial scan during an unscheduled admission. Eighteen patients had the second CT as an emergency, with a median of 237 (93–462) days between scans. For patients who died compared with the sample as a whole, the relative risk of undergoing a second scan as an emergency within the study period was 1·34 (95 per cent c.i. 1·01 to 1·77; *P* = 0·040) and in those who were obstructed the relative risk of dying from bowel perforation was 1·26 (1·07 to 1·49; *P* = 0·005).

## Discussion

Delays to cancer investigation and management are of concern both to patients and clinicians. The present results suggest that tumour volume can increase by a median of 34·1 per cent within the current NHS constitutional 62‐day standard, which brings into question this standard. Furthermore, this standard could be compromised when there is a shortage of resources for delivery of elective surgical care in patients with colorectal cancer, such as during the current COVID pandemic^37^.

To date, seven studies^19^, ^20^, ^21^, ^22^, ^23^, ^24^, ^25^ have determined tumour growth rate including a total of 177 colorectal cancers, with tumour doubling times ranging from 18 to 2593 days (*Table* *S1*, supporting information). These studies demonstrated no significant association between tumour growth rate and any change in tumour stage, nor its potential impact on treatment planning and prognosis. However, the majority of studies used barium enemas to assess tumour diameter and volume through measurement of filling defects, and were therefore limited in terms of accurate measurement of volume. Findings were also limited in terms of reproducibility owing to observed inaccuracies in tumour dimension measurements in altered views used during initial and follow‐up investigations. These studies did, however, highlight that tumour growth rate may be dependent on primary tumour location and that tumour growth rate appears to be linear^20^, ^22^, ^23^. Colorectal cancer cell type heterogeneity, and differences in genetic mutations, epigenetic regulation and the microenvironment in which tumours reside mean that predicting tumour behaviour, independent of sample size, is difficult^38^. Considering tumour growth specifically, tumour hypoxia^39^, expression of growth factors^40^, ^41^ and necrosis^42^ may be limiting factors.

An association between progression in TNM stage and tumour growth rate was demonstrated here, as expected. This study also evaluated the relationship between tumour growth rate and location (which determines non‐luminal diameter), and the findings have implications for symptom onset, time to obstruction and therefore planning of operative management. The results suggest that the more distal the tumour, the greater the median volume growth per 62 days, with a greater median absolute growth per 62 days observed in tumours that progressed from T3 to T4 compared with other stage changes. A median tumour doubling time of 211 days for the whole cohort is similar to that in the only other comparable study using CT^20^.

Eighteen of 43 patients in this cohort underwent the second CT as an emergency after the decision had been made to proceed with a non‐surgical strategy. Accepting the small sample size in this study, an all‐cause mortality rate of 81 per cent around 1 year after diagnosis is a worrying finding in this underinvestigated group. These patients are at significantly increased risk of bowel obstruction with subsequent perforation^43^. This has significant implications for the communication of prognosis with the patient, consent discussion when considering management strategy and emergency presentations^44^.

Mucinous adenocarcinomas have distinct genetic and clinicopathological features compared with non‐mucinous tumours. These tumours have previously been shown to be more frequently located in the proximal colon^45^, but there are no current data on rate of progression compared with non‐mucinous tumours. The ten mucinous tumours in the present cohort (6 right‐sided and 4 left‐sided) showed double the growth rate of non‐mucinous tumours over 62 days. If tumour growth rate could be correlated further with specific anatomical location, stage and histology, it would be possible to stratify patients in terms of risk, further prioritize management and provide a more informed consent process.

The main limitations of this study are the inherent selection bias of the study population and small sample size. The study is biased towards the inclusion of older subjects who may have more indolent tumours than a younger cohort^46^. Patient socioeconomic status and race may also affect tumour location and behaviour^46^. Inferring tumour growth rates from the observation of tumour volumes at two time points has been documented previously, but there is currently no consensus regarding the growth patterns exhibited by solid tumours or how they can be measured. The calculations applied here are based on the assumption that colorectal tumours follow a linear growth pattern, a characteristic that has yet to be established but is in line with current limited evidence (*Table* *S1*, supporting information)^47^, ^48^.

The inclusion and exclusion criteria potentially biased selection towards slow‐growing tumours in frail patients who underwent no treatment following diagnosis. This is because tumours that are large and obstructing may be subject to prompt treatment or result in morbidity. In addition, patients with smaller slow‐growing tumours that are causing minimal obstructive symptoms may be more likely to select a non‐operative treatment plan. This bias is difficult to avoid owing to the ethical considerations associated with a prospective, observational cancer growth study.

Tumour growth rate remains an important underevaluated variable in colorectal cancer, and is essential for screening, choice of management (operative *versus* non operative) and prognosis. To date, growth rate and doubling time have shown no true correlation with cancer stage or location, with limited studies pairing these parameters with histological analysis. The present results highlight particular concern regarding mucinous tumours, lesions that arise in the right colon, and tumours diagnosed as T3 that progress.

## Supporting information

**Table S1** – A Summary of Current Evidence – Radiological Colorectal Tumour Growth Patterns
