# Supplementary material for: Tumour growth rate of carcinoma of the colon and rectum: retrospective cohort study
Source: BJS Open. 2020 Sep 30;4(6):1200–7. doi: 10.1002/bjs5.50355 (PMC8370463; doi:10.1002/bjs5.50355)
Supplement: Supplementary file 1 — Table S1 – A Summary of Current Evidence – Radiological Colorectal Tumour Growth Patterns [file BJS5-4-1200-s001.doc]

**BJS5_50355**

**Tumour growth rate of carcinoma of the colon and rectum: retrospective cohort study**

J. R. Burke, P. Brown, A. Quyn, H. Lambie, D. Tolan and P. Sagar

| **Table S1 – A Summary of Current Evidence – Radiological Colorectal Tumour Growth Patterns** | | | | | | | | | | | | |
| --- | --- | --- | --- | --- | --- | --- | --- | --- | --- | --- | --- | --- |
| **Year / Country** | **Author** | **Imaging Modality** | **N** | **TNM / Dukes Sample Split** | **Tumour Location** | **Crude Measurements** | **Median Observation Time**  **(Days (Range))** | **1^st^ Investigation Median**  **Diameter (mm)** | **Linear Growth Rate (Median mm/day)** | **Volume Growth Rate**  **(cm^3^/year (Range))** | **Median Volume Doubling Time (Days (Range))** | **Findings** |
| 1963  Sweden | Welin *et. al.* (19) | Clysodrast (Bisacodyl Tannex) Enema | 20 | Not Disclosed | Tumours of the colon and rectum | Tumour Diameter | Not Disclosed | 19  (7-39) | 0.008  (0.003 – 0.025) | Not Calculated | 636  (138 - 1155) | Both linear and exponential analysis of growth rates were consistent. |
| 1974  USA | Figiel *et. al.*(21) | Barium Enema Radiographs | 18 | Not Disclosed | Not Disclosed | Tumour Diameter | Not Disclosed | Not Disclosed | Not Disclosed | Not Calculated | Not Disclosed  (116 - 478) | A polypoid tumour which does not progress in size during progress studies can be safely presumed to be benign. |
| 1974  Sweden | Ekelund *et. al.* (22) | Barium Enema Radiographs | 30 | Not Disclosed | Not Disclosed | Longitudinal Tumour Diameter | Not Disclosed | 14  (6-26) | 0.017  (0.002 – 0.067) | Not Calculated | Not Disclosed | Linear growth rates were consistent. |
| 1983  Sweden | Bolin *et. al* (23)*.* | Double Contrast Barium Enema Radiographs | 27 | A = 2  B = 7  C = 7  D = 11 | Caecum = 8  Ascending Colon = 6  Hepatic Flexure = 1  Transverse Colon = 1  Splenic Flexure = 0  Descending Colon= 0  Sigmoid Colon = 4  Rectum = 7 | Longitudinal Tumour Diameter | 1334  (122 - 2768) | 35  (10-90) | 0.083  (0.008-0.262) | Not Calculated | 130  (53-1570) | Significant correlation between poor differentiation and high linear growth rate (>0.1mm/day) was found. |
| 1984  Japan | Tada *et al.*(24) | Barium Enema Radiographs | 17 | Not Disclosed | Caecum = 1  Ascending = 1  Transverse: = 1  Descending = 2  Sigmoid = 11  Rectum = 1 | Tumour length and width | Not Disclosed | Not Disclosed | Not Disclosed | Not Disclosed | 234  (92 – 1032) | No definite correlation between doubling time and tumour histology. |
| 1996  Japan | Matsui *et. al.*(25) | Barium Enema Radiographs | 21 | Not Disclosed | Superficial Depressed Polyps: 9  Superficial Elevated (Sessile or semi-pedunculated) = 12 | Tumour Diameter | Not Disclosed | SD = 11.7mm  SE = 15.7mm | Not Disclosed | Not Disclosed | SD = 904  SE = 240 | Superficial depressed cancer develops more slowly than does elevated cancer, and this development follows a nonpolypoid growth pattern. |
| 2013  Korea | SJ Choi *et. al.* (20) | Multi-detector CT | 44 | T1=2  T2=14  T3=25  T4=3 | Caecum = 0  Ascending Colon = 15  Hepatic Flexure = 0  Transverse Colon = 2  Splenic Flexure = 0  Descending Colon =3  Sigmoid Colon = 9  Rectum = 15 | Longitudinal Tumour Diameter,  Axial Wall Thickness, Tumour Volume,  TNM Stage | 161  (47 - 628) | 36  (10-91) | 0.071  (0-0.29) | 4.65  (0.72-34.37) | 256  (18 - 2592) | Wall thickness of the tumour on initial CT demonstrated the strongest correlation with volume growth rate. |
